# Australian oncology health professionals’ knowledge, perceptions, and clinical practice related to cancer-related cognitive impairment and utility of a factsheet

**DOI:** 10.1007/s00520-022-06868-z

**Published:** 2022-02-05

**Authors:** Sharon He, Chloe Yi Shing Lim, Haryana M. Dhillon, Joanne Shaw

**Affiliations:** 1 School of Psychology, Faculty of Science, The University of Sydney, Sydney, NSW Australia; 2 Psycho-oncology Co-operative Research Group (PoCoG), School of Psychology, Faculty of Science, The University of Sydney, Sydney, NSW Australia; 3 Centre for Medical Psychology & Evidence-based Decision-making (CeMPED), The University of Sydney, Sydney, NSW Australia

**Keywords:** Cancer, Cognition, Healthcare professionals, Mixed-methods

## Abstract

**Purpose:**

Cancer-related cognitive impairment (CRCI) can have debilitating effects on cancer survivors’ quality of life. Despite this, patients often report a lack of information provided by health professionals (HPs) to assist with understanding and managing cognitive changes. This study aimed to explore Australian oncology HPs’ understanding of and clinical practice related to CRCI including the use of a Cancer Council Australia CRCI factsheet.

**Methods:**

Australian oncology HPs (medical oncologists, cancer nurses, and clinical psychologists) completed a questionnaire that assessed CRCI knowledge, prior to receiving the factsheet. Semi-structured interviews were conducted to explore their perceptions of CRCI and the factsheet. Interviews were recorded, transcribed, and analyzed using framework analysis to identify key themes.

**Results:**

Questionnaires were completed by twenty-nine HPs. Most HPs had moderate to high knowledge of CRCI, yet low knowledge of the relationship between CRCI and cancer. Twenty-six (response rate 90%) HPs; medical oncologists (*n* = 7), cancer nurses (*n* = 12), and clinical psychologists (*n* = 7), consented to be interviewed. Three main themes were identified: (1) Is CRCI impact real or over-rated?; (2) If it is important, they will tell me: identifying and responding to CRCI in clinical practice; and (3) Using a factsheet in clinical practice.

**Conclusion:**

This study’s multi-disciplinary exploration of Australian oncology HPs’ perceptions of CRCI highlighted that health professional perceptions drive CRCI discussions with patients. Further education to support clinicians to discuss CRCI is required. Consideration of the barriers and facilitators within healthcare settings is important for successful integration of the factsheet into routine care.

**Supplementary Information:**

The online version contains supplementary material available at 10.1007/s00520-022-06868-z.

## Background

Early detection of cancer and advancements in treatment have improved survival [1]. However, adverse short and long-term treatment side effects reduce cancer survivors’ quality of life [2, 3]. One such side effect is cognitive impairment, affecting up to 75% of cancer patients during chemotherapy treatment, with 35% reporting long-term side effects post treatment [4]. Changes to cognitive functioning include short-term memory loss, executive functioning impairment, decreased attention, and slower information processing [4–6]. Commonly characterized as relating to chemotherapy (“chemobrain”) [7, 8], other factors such as the cancer itself, age, genetics, hormonal therapy, or mood (e.g., depression or anxiety) can influence cognitive changes [4, 9–11]. There has been a shift towards the term CRCI, to encompass the multiple causal factors.

Qualitative studies exploring patients’ subjective experiences of CRCI indicate negative quality of life impacts [12–14]. However, survivors report inadequate information about these possible changes [15], concerns dismissed by their medical team [12], and no post-treatment assessment for cognitive changes [13]. Discussing potential changes in cognition is important as validation of symptoms, and provision of strategies to manage their impact improves overall patient functioning while reducing distress [16–19].

Few studies have explored health professionals’ (HPs) perceptions of the extent and impact of CRCI [15, 20–22]. While studies have demonstrated HPs’ awareness of CRCI in patients, there is a lack of research examining how this awareness translates into clinical practice. Given patients often feel more comfortable discussing concerns with nurses, who provide supportive care [23], it is important to explore their knowledge and perceptions of CRCI. Similarly, cancer patients may be referred to clinical psychologists for psychological distress which may exacerbate or stem from CRCI [10]; it is also important to explore their perceptions of CRCI and recommended management strategies.

To address patient information needs, the Cancer Council Australia, a national non-profit cancer support organisation, developed a CRCI factsheet [24] to provide information about CRCI and practical strategies to manage it. Widespread uptake of the factsheet requires endorsement from HPs. There is little information about how best to integrate the factsheet as a resource into routine clinical care.

The overall aim of this study was to explore Australian cancer HPs’ knowledge and perceptions about CRCI and the utility of the CRCI factsheet in clinical practice. Specifically, this study explored the following:

1. HPs’ views about the impact of CRCI on patients
2. HPs’ knowledge and current clinical practice related to CRCI and its management
3. HPs’ perceived barriers and facilitators to using the factsheet in clinical practice

## Methods

### Design

This study used a mixed-methods design. HPs completed an online questionnaire assessing knowledge of CRCI, followed by a semi-structured interview.

### Participants

Australian oncology HPs (medical oncologists, nurses, and clinical psychologists) working directly with cancer patients were purposively sampled to ensure cross-discipline representation.

### Procedure

Participants were recruited through advertisements via social media, electronic newsletters of the Psycho-oncology Co-operative Research Group, and email invitations sent to potentially eligible HPs within the authors’ professional networks. A snowballing technique was used, where participants provide names of other potentially eligible HPs [25]. Participants accessed the study via the online link and provided informed consent before completing the questionnaire.

After completing the online questionnaire, eligible participants were emailed the factsheet, prior to participating in a semi-structured telephone interview.

All interviews were audio-recorded and transcribed verbatim. A constant comparative approach was used to refine the interview questions and prompts. Recruitment continued until no new information was identified from the interviews, that is, thematic saturation was achieved.

### Measures

Participant demographic and professional characteristics were collected through the online questionnaire.

*CRCI Knowledge:* Seven multiple choice study-specific knowledge questions related to CRCI prevalence, symptoms, causes, effects, and recommended management strategies to determine HP knowledge of CRCI were developed. There were 13 criteria for CRCI symptoms, 11 causes of cognitive changes, 11 for effects of cancer, and four management strategies. One question asked whether HPs thought CRCI was real. The knowledge questions fit into three categories (low, moderate, high). Table 1 outlines the scoring criteria for each question.

**Table 1:** Level of knowledge based on identification of correct responses scoring system

	Low	Moderate	High
CRCI symptoms	0–3	4–7	8–13
Causes of CRCI	0–3	4–7	8–11
Effects of cancer	0–3	4–7	8–11
When and how many patients experience CRCI	“None of the above” or “don’t know”	Any one statistic	“All of the above”
Management strategies	0–2	3	All 4

*Qualitative Interviews:* Perceptions of CRCI, current clinical practices related to discussing and managing CRCI, and the utility of the factsheet in clinical practice were explored (Supplementary File 1).

### Data analysis

Demographic and professional characteristics and CRCI knowledge scores were descriptively summarized using Microsoft Excel.

Qualitative interviews were analyzed in Microsoft Word and Excel using a Framework approach [26]. Both deductive and inductive approaches were used to identify themes. Some codes were pre-selected based on the research questions (deductive) and other coding categories emerged through an analysis of the first four interviews using open coding (inductive). Two researchers (SH, JS) independently coded four initial transcripts to develop a thematic framework, with differences resolved through consensus. This working framework was applied to subsequent transcripts, and all data were categorized using the thematic framework and summarized within the matrix. This facilitated interpretation of the data within and across themes. Qualitative data adhered to the consolidated criteria for reporting qualitative research (COREQ) (Supplementary File 2) [27].

## Results

### Participant characteristics

Twenty-nine participants completed the initial survey, and 26 agreed to be interviewed. The median age of interview participants was 43 years (range 31–66). Consistent with clinical disciplines recruited, most HPs were female (*n* = 22); 81% had over six years clinical oncology experience. Demographic and professional characteristics are shown in Table 2.

**Table 2:** Interviewed HPs demographic and professional practice information

	Medical oncologists (MEDONC) (*n* = 7)		Clinical psychologists (CLINPSYCH) (*n* = 7)		Cancer nurses (CN) (*n* = 12)	
	Mean	SD	Mean	SD	Mean	SD
Mean age	43	12	43	10	48	11
Average patient load per week	37	16	14	7	21	7
	*n*	%	*n*	%	*n*	%
Gender						
Female	3	42	7	100	12	100
Male	4	57	0	0.0	0	0.0
Aboriginal or Torres strait islander origin						
Yes	0	0.0	0	0.0	0	0.0
No	7	100	7	100	12	100
Country of birth						
Australia	3	43	6	86	10	83
Other^a^	4	57	1	14	2	17
Language other than English at home						
Yes	3^b^	43	0	0.0	0	0.0
No	4	57	6	86	12	100
Missing	0	0.0	1	14	0	0.0
First language spoken as a child						
English	4	57	5	71	12	100
Other^c^	3	43	2	29	0	0.0
Years in oncology						
< 1 year	0	0.0	0	0.0	1	8.0
1–2 years	0	0.0	2	29	0	0.0
3–5 years	1	14	1	14	0	0.0
6–10 years	3	43	0	0.0	3	25
> 10 years	3	43	4	57	8	67
Years in current role						
< 1 year	1	14	0	0.0	1	8.0
1–2 years	2	29	3	43	0	0.0
3–5 years	2	29	0	0.0	5	42
6–10 years	1	14	0	0.0	5	42
> 10 years	1	14	4	57	1	8.0
Clinical setting						
Tertiary referral cancer center	6	86	5	71	9	75
District/local hospital	0	0.0	1	15	1	8.0
Non-inpatient cancer treatment center	0	0.0	1	15	0	0.0
Non-hospital based	1	14	0	0.0	0	0.0
Other^d^	0	0.0	0	0.0	2	17

^a^Medical oncologist: India, Sri Lanka, Taiwan. Clinical psychologist: Cyprus. Nurses: Hong Kong, Ireland

^b^Gujarati, Hindi, Mandarin

^c^Medical oncologist: Gujarati, Hindi, Mandarin. Clinical psychologist: Spanish, Greek

^d^Cancer information and support, Cancer Council

### Level of knowledge

Of the 29 HPs who completed the online survey, 27 indicated that they perceived that CRCI as a *real* condition. All HPs recognized a moderate to high number of CRCI symptoms. Compared to medical oncologists and clinical psychologists, nurses identified more CRCI symptoms. Most (*n* = 26) participants had moderate to high knowledge related to CRCI causes. However, one medical oncologist and two cancer nurses reported low knowledge. Over half of participants were found to have low knowledge (*n* = 15) of the relationship between CRCI and cancer. Most participants (*n* = 24) demonstrated high knowledge of appropriate management strategies, although five (17%) participants had only a moderate level of knowledge. A breakdown of the HP knowledge by discipline is shown in Table 3.

**Table 3:** HP scores for CRCI knowledge questions

	Medical oncologist (*n* = 8)		Clinical psychologist (*n* = 7)		Cancer nurse (*n* = 14)	
	*n*	%	*n*	%	*n*	%
CRCI real?						
Yes	8	100	6	86	13	93
Not sure	0	0.0	1	15	1	7.0
No	0	0.0	0	0.0	0	0.0
CRCI symptoms						
Low	0	0.0	0	0.0	0	0.0
Mid	4	50	4	57	2	14
High	4	50	3	43	12	86
Causes of CRCI						
Low	1	13	0	0.0	2	14
Mid	6	74	2	28	5	36
High	1	13	5	72	7	50
Effect of cancer						
Low	5	63	4	58	6	43
Mid	2	25	1	14	2	14
High	1	12	2	28	6	43
When and how many patients experience CRCI						
Low	0	0.0	0	0.0	1	7.0
Mid	5	63	3	43	4	14
High	3	37	4	57	9	79
Management strategies						
Low	0	0.0	0	0.0	0	0.0
Mid	2	26	2	28	1	7.0
High	6	74	5	72	13	93

### Qualitative data

**Fig. 1:**
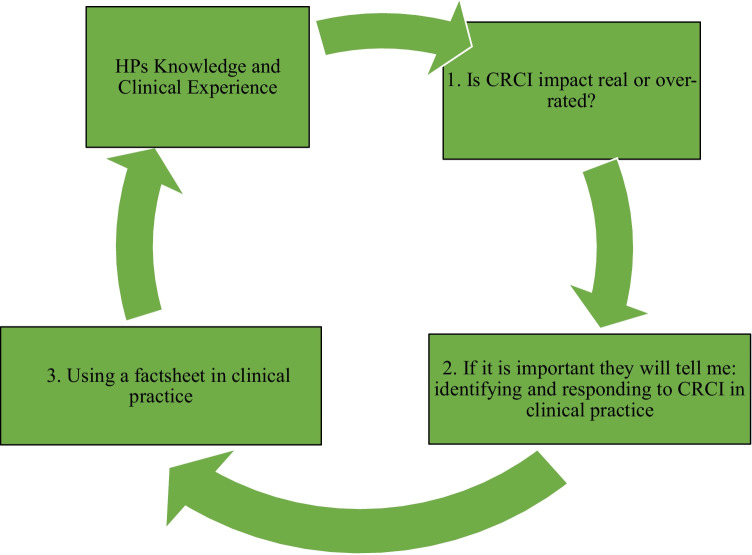
Graphical representation of relationship between themes

Median interview length was 17 min (range 11–30). Recruitment concluded when thematic saturation was reached (22 participants), although four additional interviews were conducted to confirm saturation. From the 26 interviews, three main themes emerged: (1) Is CRCI impact real or over-rated?; (2) If it is important, they will tell me: identifying and responding to CRCI in clinical practice; and (3) using a factsheet in clinical practice. Quotes are identified by participant ID and profession (e.g., HP01_MEDONC). Figure 1 provides a graphical representation of the relationship between themes.

#### 1. CRCI impact-real or over-rated?

Many HPs viewed CRCI as an important aspect of the patient’s cancer experience. HP views derived from both empirical knowledge and clinical experience. Despite the perceived importance, the majority of HPs reported that CRCI was “*still evolving…not well defined*” (HP11_MEDONC). Interestingly, some cancer nurses perceived that CRCI was not adequately explored and recognized enough by other HPs, while only a few medical oncologists felt it was under recognized by clinicians.

*"I think it’s immense and probably underestimated by the medical profession, by healthcare institutions and they probably underestimate the impact of this on patients …"* (HP03_CN)

Based on their clinical experience, most HPs perceived the impact of CRCI on patient’s daily lives was variable, from quite mild where it did not seem to affect patient’s daily lives, to significant and debilitating.

*"It really varies because it’s [a] very variable condition… where people just go, “Oh I just feel a little bit foggy,” to people going, “Oh I keep forgetting stuff,” … some people say, “I’ve had to cut down on the amount of work because I just can’t concentrate anymore.”"* (HP12_CN)

However, some HPs perceived CRCI to be less common, with most medical oncologists stating only a small proportion of their patients’ actively self-report symptoms of CRCI.

*"I don’t actively ask patients so I would probably estimate ten percent to actively report it."* (HP25_MEDONC)

Similarly, most clinical psychologists did not receive referrals specifically for CRCI, but it was a secondary problem or concern raised during sessions.

*"I think perhaps some of our referrers will notice distress first and then they will add, “Oh, cognitive impairment” or “chemobrain” as well…"* (HP13_CLINPSYCH)

*"…They're rarely referred specifically for memory problems; it’s usually other issues and then when we’re talking I ask about things like that and that’s when it comes out…"* (HP07_CLINPSYCH)

HPs’ perceptions of which patients were likely to experience CRCI varied. Some HPs perceived different cancer types experienced greater symptomology; others noted patients who undergo multiple therapies reported greater CRCI. There were also perceived differences related to age, with elderly patients considered at greater risk. All HPs highlighted patients report symptoms during and after treatment.

*"Well I guess we tend to hear more from women who have been through gyny or breast cancer treatments but I hear it from bowel patients, men with prostate cancer and of course it’s interesting for me to be aware that it’s just not chemotherapy that can cause these cognitive changes for people, that the cancer itself seems to play a role …"* (HP14_CN)

#### 2. If it is important, they will tell me: identifying and responding to CRCI in clinical practice

Despite hypothetical acknowledgement of CRCI, few HPs reported discussing CRCI with patients. Most CRCI conversations were initiated by patients rather than clinical teams. HPs perceived that patients experiencing CRCI would raise their concerns during consultations and highlighted that there was little time in consultations to ask every patient. However, several HPs reported that they would initiate CRCI-related conversations either directly as it was part of their role, or if symptoms were reported indirectly by patients in general distress screening assessments. Similarly, nurses stated CRCI would be *“briefly touched on”* (HP17_CN). Interestingly, one HP (HP20_MEDONC) stated that they would not discuss CRCI with their patients as it would be “*counterproductive*,” as mentioning the possibility of CRCI to patients would cause them to “*magnify to an unrealistic level*” (HP20_MEDONC)*.*

*"…sometimes the patient or the family members might say “oh mum’s a little bit more forgetful- than usual since starting treatment or since the diagnosis"* (HP17_CN)

"… *patients will bring it up themselves…they will say that they are feeling tired, feeling very slow, having difficulty remembering certain things because I think that’s something the patients worry about a lot…so they will tend to usually bring it up themselves*… (HP21_MEDONC)

A few HPs reported barriers to CRCI conversations such as uncertainty around managing CRCI, lack of resources and time to address cognitive changes reduce the likelihood of them raising it. Similarly, medical oncologists reported a lack of a validated screening or assessment measure for CRCI meant they did not routinely discuss CRCI.

*"…We don’t really know how to ask about it, how to measure it and secondly I suppose we feel responsible for it and in a way it’s like um say “have you got this because the chemotherapy may have caused it but I can’t do anything about it”. I think so I think it’s not asked about enough because it’s hard to talk about, hard to assess and hard to fix"* (HP18_MEDONC)

HPs agreed screening for CRCI would be helpful, but were wary about potential barriers to implementing screening, such as lack of resources and time. Some HPs were concerned about the potential patient burden of additional screening, particularly without a clear referral pathway for treatment. Many stated that they would prefer CRCI screening to be integrated into current screening protocols.

*"I guess it would be useful, I guess just considering what’s the extra burden on the patient and that there’s often quite a lot of screens they do…"* (HP15_CLINPSYCH)

"…*I shouldn’t screen for something unless I have the facilities to deal with what I find"* (HP18_MEDONC)

"…*I do think it should be included as part of a good psychosocial screen so yes. But not as a standalone screen, no*." (HP10_CN)

A few medical oncologists stated that the lack of a validated measure to determine the level of CRCI severity made it difficult for them to objectively determine how impaired the patient was and thus indicated preference for an “*expert to give [HPs] the best validated short question*” (HP20_MEDONC).

HPs management of CRCI seemed to be mediated by their perceived role in discussing CRCI with patients. A few nurses stated that it was part of their role to discuss how best to manage CRCI, while others discuss CRCI management if raised by patients themselves. Similarly, most medical oncologists reported discussing CRCI management when raised by patients themselves. While majority of psychologists did not receive referrals for CRCI as primary issue, many would discuss and provide management strategies if this was raised during consultations.

"… *So it comes up you know I either ask them directly or they've brought it, they’ve initiated that um but that’s [management] often…part of the focus of the service that I provide"* (HP03_CN)

*"Yeah if they come up…a lot of people will say…”oh you know I can’t remember that today, oh did we say that…” and you know they will attribute it to chemobrain so then it comes up and that then open the doors to do some work around it"* (HP13_CLINPSYCH)

The most common strategy to manage CRCI was to normalize the experience and provide reassurance. Other strategies reported included compensatory strategies such as “writing things down, keeping a diary…so using the memory aid” (HP07_CLINPSYCH).

*"…It’s almost starting with the validation of, “It’s okay to feel that way”"* (HP04_CN)

*"Yeah, well there’s the hard bit… so most of what I’m doing is reassurance…"* (HP18_MEDONC)

#### 3. Factsheet use in clinical practice

Most HPs stated that they would use the factsheet in their consultations *only* if patients reported CRCI. A few stated that they would display the factsheet in their waiting rooms but would *not provide* the factsheet to avoid burdening the patient with too much information. Some medical oncologists perceived the factsheet as a cue for them to discuss the topic with patients. Several clinical psychologists stated that the factsheet would be beneficial if incorporated into their treatment plan as a summary of CRCI discussions. In contrast, many cancer nurses felt the factsheet should be provided as a standard resource at the beginning of treatment so patients are aware of potential changes to their cognitive functioning, with one HP commenting it would be a “*beneficial factsheet used across a continuum of care*” (HP01_CN)*.*

*"I think [I’m] probably more likely if I have the factsheet to be able to ask more patients and volunteer it to more patients"* (HP22_MEDONC)

*"…If I knew that I would have it with me when I went into sessions. If I didn’t know and it came up in sessions, then as long as I knew that we had it in a resource area then as I walked somebody out, I would take them and give it to them…"* (HP13_CLINPSYCH)

Participants highlighted the need for training/education about CRCI to build confidence in initiating discussion with patients. Some HPs highlighted the need for a more detailed factsheet similar to the current one but targeting HPs rather than patients.

*"I think the factsheet that you’ve provided is obviously available to patient[s] and perhaps a similar sort of factsheet for clinicians would be useful..."* (HP18_MEDONC)

## Discussion

This study aimed to explore Australian oncology HPs’ perceptions and knowledge about CRCI and the utility of a CRCI factsheet in clinical practice. The results suggested that HPs’ views and perceptions of CRCI, driven by their knowledge and clinical experience, influenced the extent to which cognitive impairment was discussed. The results also indicated that uptake of the factsheet into clinical practice requires consideration of barriers within the healthcare system.

Extending previous research, we identified that HPs’ views and perceptions influenced their clinical practice. HPs with greater knowledge and experience of CRCI were more likely to recommend management strategies to patients. This finding was consistent across disciplines. Research in the wider clinical context has suggested health practitioners’ knowledge often influences their clinical behavior [28, 29]. This implies that for practice change to occur, HPs need education about CRCI to facilitate discussions and deliver appropriate strategies for patients.

Surprisingly, there were differences between HPs’ recognition of CRCI, presented in the study-specific knowledge questions, and information volunteered in the interviews. In general, cancer nurses were able to identify more CRCI symptoms than medical oncologists and clinical psychologists. This is consistent with research suggesting greater disclosure of concerns between patients and nurses compared to doctors [23]. While most participants demonstrated good knowledge CRCI’s causes, half the survey participants had low knowledge concerning the relationship between cancer and cognition. Given that studies have reported the cancer itself may affect cognitive functioning [9, 30, 31], this implies that patients’ concerns of CRCI may not be adequately recognized by HPs due to a perception of treatment-related cognitive impairments.

Interestingly, there were inconsistencies between HPs’ recognition of management strategies for CRCI and the provision of strategies in clinical practice. Qualitatively, participants reported using few of the strategies identified in the knowledge questionnaire, suggesting that HPs’ knowledge of CRCI has not translated into clinical practice.

Similar to previous research [15, 22], most HPs did not discuss CRCI unless it was raised by patients. HPs were reluctant to initiate discussions around CRCI as CRCI is not currently clearly defined and HPs expressed the need for more information to guide patients and clinicians. Given most cancer patients want information about diagnosis, treatment and management strategies [32]**,** it is crucial that HPs have and can provide information and strategies to manage CRCI.

### Uptake of CRCI factsheet in routine clinical care

An important consideration when implementing the factsheet into clinical practice is its feasibility and acceptability. Based on the Promoting Action on Research Implementation in Health Services (PARiHS) framework, successful implementation of interventions requires consideration of three domains: (1) evidence, (2) context, and (3) facilitation [33]. Successful implementation of interventions into clinical practice occurs when there is high evidence supporting the proposed intervention and perceived sufficient clinical experience in the area, the context of the clinical practice is responsive to change, and the intervention has facilitators or champions [34]. It is important to identify and address the barriers and facilitators perceived by HPs in using the factsheet in clinical care.

A need for evidence supporting intervention is important for successful implementation [33]. Most HPs’ perceptions of CRCI were based on their clinical experience with many commenting that the lack of evidence regarding cause, identification, and treatment was a barrier to discussing CRCI with patients. HPs viewed the factsheet as a tool to provide evidence and management strategies for CRCI, facilitating HP-driven CRCI discussion, with some suggesting for a CRCI factsheet targeted towards HPs.

The PARiHS framework also highlights the importance of context when implementing interventions [33]. Pressure within the healthcare system is a commonly reported barrier by staff working in the hospital context [35]. Furthermore, timing of when to provide the factsheet is important to address patients’ information needs and manage anxiety [36]. HPs recognized time pressures and lack of resources as barriers to factsheet use and would only provide the factsheet if patients reported CRCI symptoms. Some HPs were also concerned that provision of factsheet would overwhelm patients with information, while cancer nurses felt that the factsheet should be provided at the beginning of treatment. Our companion study, evaluating the factsheet with cancer patients, concluded that it should be given early after diagnosis and again throughout the treatment and follow-up trajectory [37].

The last component of the PARiHS framework refers to an individual who champions or facilitates the implementation [33]. While characteristics of facilitators were not discussed, there seemed to be a need for leadership and champions amongst the HPs to facilitate use of the factsheet due to their perceived role in providing information about CRCI to patients. Several cancer nurses stated acting in supportive roles rather than actively identifying patients with CRCI, while many clinical psychologists reported no referrals for CRCI or CRCI as secondary to the primary reason for referral. Therefore, organizational support for practice change is needed to enable multi-disciplinary agreement and reinforce new routine practices and actively integrate the factsheet into clinical practice.

## Limitations

This study is not without limitations. Most participants recruited were female practitioners and primarily based in tertiary referral cancer centers. Thus, the results of the study may not be generalizable to other HPs who are based in different clinical settings such as private or regional centers. Participant recruitment was dependent on HPs’ interest; therefore, HPs interviewed could have perceived CRCI a prominent issue or had greater awareness of CRCI. Therefore, there could be selection bias and data may not represent the views of all HPs.

## Conclusion

This study provides important insight into HPs’ perceptions and management of CRCI. HPs’ awareness of CRCI depended on their clinical experience and knowledge of CRCI. HPs with greater knowledge and wider clinical experience were more likely to discuss CRCI with patients. In general, cancer nurses provided reassurance and a few practical strategies, while medical oncologists provided validation of symptoms to patients. Many HPs expressed the lack of knowledge and clarity around the underlying mechanisms for CRCI rendered them reluctant to initiate discussion with patients. Provision of education/training and clarity around roles in the clinical setting is crucial for successful uptake of the factsheet into routine care.

## Coding availability

Not applicable.

## Supplementary Information

Below is the link to the electronic supplementary material.

Supplementary file1 (PDF 281 KB)

Supplementary file2 (PDF 278 KB)

## Data Availability

Data and material are available from the corresponding author upon reasonable request.
