# Supplementary material for: Australian oncology health professionals’ knowledge, perceptions, and clinical practice related to cancer-related cognitive impairment and utility of a factsheet
Source: Support Care Cancer. 2022 Feb 5;30(6):4729–38. doi: 10.1007/s00520-022-06868-z (PMC9046357; doi:10.1007/s00520-022-06868-z)
Supplement: Supplementary file 1 — Supplementary file1 (PDF 281 KB) [file 520_2022_6868_MOESM1_ESM.pdf]

Australian oncology health professionals' knowledge, perceptions and clinical practice related to cancer-related cognitive impairment and utility of a factsheet

Supportive Care in Cancer

Sharon He[1,2], Chloe Yi Shing Lim[1,3], Haryana M Dhillon[1,2,3], Joanne Shaw [1,2]

1. School of Psychology, Faculty of Science, The University of Sydney, Sydney, NSW, Australia

2. Psycho-oncology Co-operative Research Group (PoCoG), School of Psychology, Faculty of Science, The University of Sydney, Sydney, NSW, Australia

3. Centre for Medical Psychology & Evidence-based Decision-making (CeMPED), The University of Sydney, Sydney, NSW, Australia

Corresponding author: Joanne Shaw, joanne.shaw@sydney.edu.au

Supplementary File 1: Semi-structured Interview Guide.

1. Did you have time to read the factsheet?
   If Yes: continue to question 2.
   If No: A number of questions relate directly to the factsheet; can we organize another time to call to give you time to read the factsheet?
2. Have you seen the factsheet before I sent it to you?

Thought on Cancer-Related Cognitive Impairments

3. In your experience, what do you think is the impact of cognitive impairment (CI) for patients?

Prompts: How does it impact patient's functioning in their daily lives?
  How important do you think it is to explore patients experience of cognitive impairment?
  Are there particular patient groups who are more affected or prone to cognitive impairment?

3a. If they say they don't believe in CI or not sure whether CI is a side effect:
  Do your patients ever mention it to you? (Although clinically you don't see it as an important issue, what do you think is the patient's perspective on it?)
    If Yes: *How do you discuss their concerns with them*?

Questions specific to Clinical Psychologists
4. Do you receive referrals for cognitive impairment?
5. What's the relationship between cognitive impairment and emotional distress in patients you see?
6. How do you manage cognitive impairment in patients you see? (*Discuss strategies)*
  Prompts: Self-management strategies?
    Provide psychoeducation/support?
    Don't provide support?
7. How many patients are coming to you because of cognitive impairment?
8. How likely would it be for you to refer patients for neuropsychological assessments?
  a. How do you decide who to send?

9. You mentioned that you see about ___ patients per week. Of these patients, how many would you say report experiencing cognitive impairment?
10. If a patient is concerned about cognitive impairment or reports symptoms of cognitive impairment, how do you manage this?

Prompts: Referral? If so, who to? Why?
  Self-management strategies?
  Provide psychoeducation/support?
  Don't provide support?

11. Do you discuss cancer-related cognitive impairments with your patients?
  If Yes: Who usually brings up the topic of cancer-related cognitive impairments?
    If Patients:
      a. What sort of changes in their cognitive functions do patients talk about?
      b. Who tells you about it? *(i.e. patients themselves, carers)*
    If HP brings up the topic*:*
      a. What do you typically discuss with patients?

b. How do you decide who to discuss it with? Why? *(to figure out whether there is a bias about health literacy, is it due to age/cultural factors/gender/treatment?)*
c. When do you usually talk about changes in cognitive functioning? *(i.e. before treatments, during treatment or after treatment)*

11a. If they don't discuss cognitive changes with patients: Is there any reason?
a. What are the barriers?
If mention priming: *What are your thoughts on priming patients about cancer-related cognitive impairments to validate their experience?*
If mention lack of guidelines/uncertainty: *What do you think should be used to help you overcome the uncertainty? (e.g., conferences to educate HP on cancer-related cognitive impairments)*

12. Do you advise your patients on how to manage these symptoms? (if not answered already)
If Yes: *Do you provide information resources to patients? If so how? If not, why?*
If No: *Why?*

Thoughts on the Factsheet

13. Having read the factsheet, what are your thoughts on the factsheet?
14. Do you think the information is accurate and evidence-based?
15. Is the information pitched at the right level for your patients?
16. What are your thoughts on the factsheet:
a. in respects to the level of content?
Prompts: Is it too difficult, is there is too much content/information?
b. in respects to the clarity of information presented?
Prompts: Is it clear/does it make sense?
17. Are there aspects that could be improved, or you think are missing?
18. Would you use the factsheet?
If Yes: *how?*
If No: *why?*
If they don't believe in CI and say factsheet is okay: *When you are talking about strategies you use, do you talk specifically about adjusting routine/what kind of strategies do you use?*
19. Would you provide the factsheet to patients or use the factsheet in your consultations?
If Yes: *how do you think you would use the factsheet (are there specific patient groups? Etc.)*
If No: *why?*
20. Do you find factsheets developed by Cancer Council reliable?

Thoughts on Screening tools for Cancer-Related Cognitive Impairments

21. Do you think we should screen for cognitive problems?
22. Are you currently screening? If so how?
23. Are you aware of any other screening tools?

Further Thoughts

24. That's all the question that I had, is there anything else that you would like to ask or comment on about the factsheet?
