# Supplementary material for: Australian oncology health professionals’ knowledge, perceptions, and clinical practice related to cancer-related cognitive impairment and utility of a factsheet
Source: Support Care Cancer. 2022 Feb 5;30(6):4729–38. doi: 10.1007/s00520-022-06868-z (PMC9046357; doi:10.1007/s00520-022-06868-z)
Supplement: Supplementary file 2 — Supplementary file2 (PDF 278 KB) [file 520_2022_6868_MOESM2_ESM.pdf]

Australian oncology health professionals' knowledge, perceptions and clinical practice related to cancer-related cognitive impairment and utility of a factsheet

Supportive Care in Cancer

Sharon He[1,2], Chloe Yi Shing Lim[1,3], Haryana M Dhillon[1,2,3,] Joanne Shaw [1,2]

1. School of Psychology, Faculty of Science, The University of Sydney, Sydney, NSW, Australia

2. Psycho-oncology Co-operative Research Group (PoCoG), School of Psychology, Faculty of Science, The University of Sydney, Sydney, NSW, Australia

3. Centre for Medical Psychology & Evidence-based Decision-making (CeMPED), The University of Sydney, Sydney, NSW, Australia

Corresponding author: Joanne Shaw, joanne.shaw@sydney.edu.au

Supplementary File 2: COREQ checklist.

Consolidated criteria for reporting qualitative studies (COREQ) guidelines checklist: 32-item checklist.

Topic	Item	Guide questions/description	Page Number/Response
**Domain 1: Research team and reflexivity**			
Personal characteristics			
Interviewer/facilitator	1	Which author/s conducted the interview or focus group?	All interviews were conducted by SH.
Credentials	2	What were the researcher's credentials? E.g. PhD, MD	SH: Honours student in psychology.<br>JS: PhD
Occupation	3	What was their occupation at the time of the study?	SH: was an honours student supervised by JS.<br>JS: Senior research fellow, Executive Director, Psycho-Oncology Co-operative Research Group
Gender	4	Was the researcher male or female?	Female.
Experience and training	5	What experience or training did the researcher have?	Prior to conducting interviews, SH attended a qualitative research workshop and completed two practice interviews with JS and HD.
Relationship with participants			
Relationship established	6	Was a relationship established prior to study commencement?	The interviewer did not know the participants prior to the study.
Participant knowledge of the interviewer	7	What did the participants know about the researcher? e.g. personal goals, reasons for doing the research	The participants did not know anything about the interviewer (SH), except that the study was for SH's honours project.

Interviewer characteristics	8	What characteristics were reported about the interviewer/facilitator? e.g. Bias, assumptions, reasons and interests in the research topic	Throughout data collection, the researcher recorded notes post-interview and transcription to ensure transparency and to mitigate any potential influence during data collection.
**Domain 2: study design**			
Theoretical framework			
Methodological orientation and Theory	9	What methodological orientation was stated to underpin the study? e.g. grounded theory, discourse analysis, ethnography, phenomenology, content analysis	5; "*Qualitative interviews were analysed in Microsoft Word and Excel using a Framework approach [26].*"
Participant selection			
Sampling	10	How were participants selected? e.g. purposive, convenience, consecutive, snowball	4; "*…purposively selected to ensure cross-discipline representation"* and "*snowballing technique was used*".
Method of approach	11	How were participants approached? e.g. face-to-face, telephone, mail, email	4; "*Participants were recruited through advertisements via social media, electronic newsletters of the Psycho-oncology Co-operative Research Group and email invitations sent to potentially eligible HPs within the authors' professional networks*".
Sample size	12	How many participants were in the study?	5; "*Twenty-nine participants completed the initial survey and 26 participants agreed to be interviewed.*"
Non-participation	13	How many people refused to participate or dropped out? Reasons?	5; "*Twenty-nine participants completed the initial survey and 26 participants agreed to be interviewed.*"
Setting			

Setting of data collection	14	Where was the data collected? e.g. home, clinic, workplace	Telephone interviews conducted either in private office booth or private room at home.
Presence of non-participants	15	Was anyone else present besides the participants and researchers?	No.
Description of sample	16	What are the important characteristics of the sample? e.g. demographic data, date	Table 2.
Data collection			
Interview guide	17	Were questions, prompts, guides provided by the authors? Was it pilot tested?	Supplementary File 1.
Repeat interviews	18	Were repeat interviews carried out? If yes, how many?	No.
Audio/visual recording	19	Did the research use audio or visual recording to collect the data	4; "*All interviews were audio-recorded and transcribed verbatim.*"
Field notes	20	Were field notes made during and/or after the interview or focus group?	SH recorded reflective notes after each interview.
Duration	21	What was the duration of the interviews or focus group?	6; "*Median interview length was 17 minutes (range 11-30).*"
Data saturation	22	Was data saturation discussed?	6; "*Recruitment concluded when thematic saturation was reached (22 participants), although an additional four interviews were conducted to confirm saturation.*"

Transcripts returned	23	Were transcripts returned to participants for comment and/or correction?	No.
**Domain 3: analysis and findings**			
Data analysis			
Number of data coders	24	How many data coders coded the data?	5; "*Two researchers (SH, JS) independently coded four initial transcripts to develop a thematic framework, with differences resolved through consensus.*"
Description of the coding tree	25	Did authors provide a description of the coding tree?	5; "*This working framework was applied to subsequent transcripts, and all data were categorized using the thematic framework and summarized within the matrix. This facilitated interpretation of the data within and across themes.*"
Derivation of themes	26	Were themes identified in advance or derived from the data?	5; "*Both deductive and inductive approaches were used to identify themes. Some codes were pre-selected based on the research questions (deductive) and other coding categories emerged through an analysis of the first four interviews using open coding (inductive).*"
Software	27	What software, if applicable, was used to manage the data?	5; "*Qualitative interviews were analysed in Microsoft Word and Excel…*"
Participant checking	28	Did participants provide feedback on the findings?	No, but a one-page lay summary of the results was emailed to participants on study completion.
Reporting			
Quotations presented	29	Were participant quotations presented to illustrate the themes / findings? Was each quotation identified? e.g. participant number	6-11; Results section.

Data and findings consistent	30	Was there consistency between the data presented and the findings?	6-11; Results section.
Clarity of major themes	31	Were major themes clearly presented in the findings?	6-11; Results section.
Clarity of minor themes	32	Is there a description of diverse cases or discussion of minor themes?	6-11; Results section.
